# Problems persist in reporting of methods and results for the WOMAC measure in hip and knee osteoarthritis trials

**DOI:** 10.1007/s11136-018-1978-1

**Published:** 2018-09-18

**Authors:** B. Copsey, J. Y. Thompson, K. Vadher, U. Ali, S. J. Dutton, R. Fitzpatrick, S. E. Lamb, J. A. Cook

**Affiliations:** 1 Nuffield Department of Orthopaedics, Rheumatology and Musculoskeletal Sciences (NDORMS), Botnar Research Centre, University of Oxford, Windmill Road, Headington, Oxford, OX3 7LD UK; 2 Nuffield Department of Population Health, University of Oxford, Richard Doll Building, Old Road Campus, Oxford, OX3 7LF UK

**Keywords:** WOMAC, Osteoarthritis, Randomised trial, Reporting

## Abstract

**Purpose:**

The Western Ontario and McMaster Universities Arthritis Index (WOMAC) is a commonly used outcome measure for osteoarthritis. There are different versions of the WOMAC (Likert, visual analogue or numeric scales). A previous review of trials published before 2010 found poor reporting and inconsistency in how the WOMAC was used. This review explores whether these problems persist.

**Methods:**

This systematic review included randomised trials of hip and/or knee osteoarthritis published in 2016 that used the WOMAC. Data were extracted on the version used, score range, analysis and results of the WOMAC, and whether these details were clearly reported.

**Results:**

This review included 62 trials and 41 reported the WOMAC total score. The version used and item range for the WOMAC total score were unclear in 44% (*n* = 18/41) and 24% (*n* = 10/41) of trials, respectively. The smallest total score range was 0–10 (calculated by averaging 24 items scored 0–10); the largest was 0–2400 (calculated by summing 24 items scored 0–100). All trials reported the statistical analysis methods but only 29% reported the between-group mean difference and 95% confidence interval.

**Conclusion:**

Details on the use and scoring of the WOMAC were often not reported. We recommend that trials report the version of the WOMAC and the score range used. The between-group treatment effect and corresponding confidence interval should be reported. If all the items of the WOMAC are collected, the total score and individual subscale scores should be presented. Better reporting would facilitate the interpretation, comparison and synthesis of the WOMAC score in trials.

**Electronic supplementary material:**

The online version of this article (10.1007/s11136-018-1978-1) contains supplementary material, which is available to authorized users.

## Introduction

A lack of clarity and transparency in how outcome measurement tools are used in clinical research can make it difficult to interpret study results [1, 2]. It can be hard to decipher whether a clinically meaningful effect has occurred when it is unclear how the outcome scores have been calculated. Additionally, inconsistency in how an outcome measure is used can make it more difficult to compare the results with previous research findings. A systematic review published in 2012 identified issues of inconsistency and poor reporting of the methods and results of the WOMAC measure in knee osteoarthritis trials, including poor reporting of the type of scale used and how the WOMAC measure was administered [3].

The Western Ontario and McMaster Universities Arthritis Index (WOMAC) is a patient-reported outcome measure for the assessment of lower limb osteoarthritis [4]. The WOMAC measure has been used for decades [5–7] and is one of the most commonly used outcome measures in hip and knee osteoarthritis research [8–10]. The WOMAC measure has been used in clinical trials to evaluate the efficacy of surgical treatments [11, 12], medicinal and biological products [13, 14], devices [15] and physical therapies [16]. The WOMAC has been recommended as a trial endpoint by the Food and Drug Administration (FDA) [13] and is noted as a potential measure for efficacy in recommendations for updates of FDA and European Medicines Agency (EMA) guidance [17–19] and other working groups [20, 21]. In addition, the WOMAC has been recommended as one of the highest-performing outcome measures for knee and hip osteoarthritis, in terms of reliability, validity, responsiveness and interpretability [22–24]. The WOMAC is often used as a comparator to assess the measurement properties of other outcome measures [25, 26]. However, there is variation in how the WOMAC outcome score is collected and calculated.

The WOMAC is composed of 24 items over 3 subscales (5 for pain, 2 for stiffness and 17 for physical function). Participants rate their difficulty for each item, for example, their pain level going up and down stairs or their difficulty rising from a chair. The seminal paper did not provide the wording for the individual questions used in the questionnaire. A user guide for the WOMAC is not freely available. The user guide includes information on how the WOMAC was derived, calculation of scores and specific clinimetric and statistical issues. To obtain the user guide, users (researchers or clinicians) are required to submit a request to the developer of the WOMAC via the website http://www.womac.org, including their personal details and information on the intended use of the WOMAC measure [27]. As well as being translated into over 90 languages, different versions of the WOMAC measure exist [28]. Items can be rated on a five-level Likert scale (no difficulty to extremely difficult) or using a 0–100 mm visual analogue scale (VAS) or an 11-point numerical rating scale (NRS from 0 to 10). These different approaches mean that the range of the WOMAC scores can be unclear if it is not explicitly stated [3]. There are also variations in how item scores are combined, for example, using the total or average of the items. The effect on the total score range of the different options for scoring and combining individual items of the WOMAC is shown in Table 1.

**Table 1:** Score range for the WOMAC measure using different versions and methods to combine 24 individual item scores

	Combine 24 item scores using		
	Total	Average	Percentage
Version used for item scores			
Likert scale (item 0–4)	0–96	0–4	0–100
NRS (item 0–10)	0–240	0–10	0–100
VAS (item 0–100)	0–2400	0–100	0–100

A lack of clarity in the use and scoring of the WOMAC, including the version used and the score range, can hinder the interpretation of study results. It is difficult to interpret the importance of a 10 point difference between the treatment groups when it is unclear whether this relates to a score range of 0–96 or 0–2400. This also causes problems when combining and comparing results across different trials if it is unclear whether trials have measured and combined the items of the WOMAC differently.

The statistical analysis methods used and how the trial results are reported can also make it more difficult to compare and synthesise results across different trials. The use of different techniques, dichotomisation of continuous measures, adjusting for different covariates or how missing data are handled can impact on the overall treatment effect. Therefore, it is important to be transparent in the methodology used to analyse trial outcomes. Poor reporting of the results of the analysis can also make it more difficult to interpret the trial results. For example, reporting only the *p* value from the analysis and omitting the between-group mean difference mean that the reader cannot assess the clinical significance of the treatment effect on the original scale of the outcome measure.

The previous review that found poor reporting on how the WOMAC was used included trials of physical therapies published prior to 2010 [3]. Since then, there have been considerable efforts to improve the reporting of clinical trials [29]. To our knowledge, no previous review has examined the use and scoring of all of the WOMAC subscales (including pain, stiffness and function) or the statistical analysis of the WOMAC measure.

This review aims to examine the clarity and consistency of the scoring, analysis and reporting of the WOMAC measure and its subscales in two-arm randomised trials of hip and/or knee osteoarthritis published in 2016.

## Methods

### Identification of studies

This review utilised a systematic review which identified a cohort of osteoarthritis trials published in 2016 [30].

In the previous review, the cohort of trials were identified by searching seven databases for clinical trials of osteoarthritis from inception to 31 March 2017: Medline, Cochrane Central Register of Controlled Trials (CENTRAL), CINAHL, EMBASE, AMED, PsycINFO and PEDro. Searches were limited to trials published in 2016. An example of the search strategy developed for the previous review of osteoarthritis trials is given in Online Resource 1 [30].

### Selection of studies

#### Eligibility criteria for the cohort of trials

The cohort of trials included in the previous review were two-arm parallel-group randomised controlled trials in a sample of people living with hip and/or knee osteoarthritis published in 2016. The cohort did not include pilot or feasibility studies, since the main outcomes could relate to, for example, feasibility of recruitment, rather than measuring the efficacy of the intervention.

#### Additional screening criteria

We restricted the eligibility to include only those trials that measured the WOMAC or one of its subscales (pain, stiffness or physical function) as a trial outcome, whether primary or secondary. Trials that only measured the WOMAC at baseline were excluded since we expected these trials to report fewer details on the use of the WOMAC when it was not used as a trial outcome to measure treatment efficacy. Eligibility screening was conducted independently by pairs of reviewers.

### Data extraction

Data extraction on study characteristics included the study design, population, sample size and follow-up assessment time points. To further describe the trial samples, data were extracted on the baseline demographics for the WOMAC (e.g. mean score) and whether the WOMAC was used to restrict eligibility to participate in the trial.

For the WOMAC measure and its subscales, data were extracted on the version of the WOMAC used (VAS, NRS or Likert scale) and the scoring system (range of individual items and overall scale or subscale). It was noted when these details were unclear or not reported.

Data on the statistical methods used to analyse the WOMAC, including covariate adjustment and dichotomisation of the WOMAC score, were also extracted and summarised. This would impact the effect estimates reported for the WOMAC score. Summarising this information on the analysis of the WOMAC will allow future trials to utilise consistent methodology to facilitate the comparison of trials results. Data were extracted on how trials examined the impact of missing data on the WOMAC to explore the likelihood of bias in the treatment effects.

Finally, data were extracted on how the results of the WOMAC measure were reported, for example, whether and how the within-group WOMAC scores and the between-group treatment effect were presented. The reporting of the results of the WOMAC will influence the ease with which trial results can be interpreted, compared and combined together.

### Data synthesis

Categorical data were summarised using the number and proportion within each category. For continuous outcomes, data were summarised using the median and interquartile range. Data were summarised separately for the WOMAC total score and each individual subscale.

## Results

### Characteristics of included studies

**Fig. 1:**
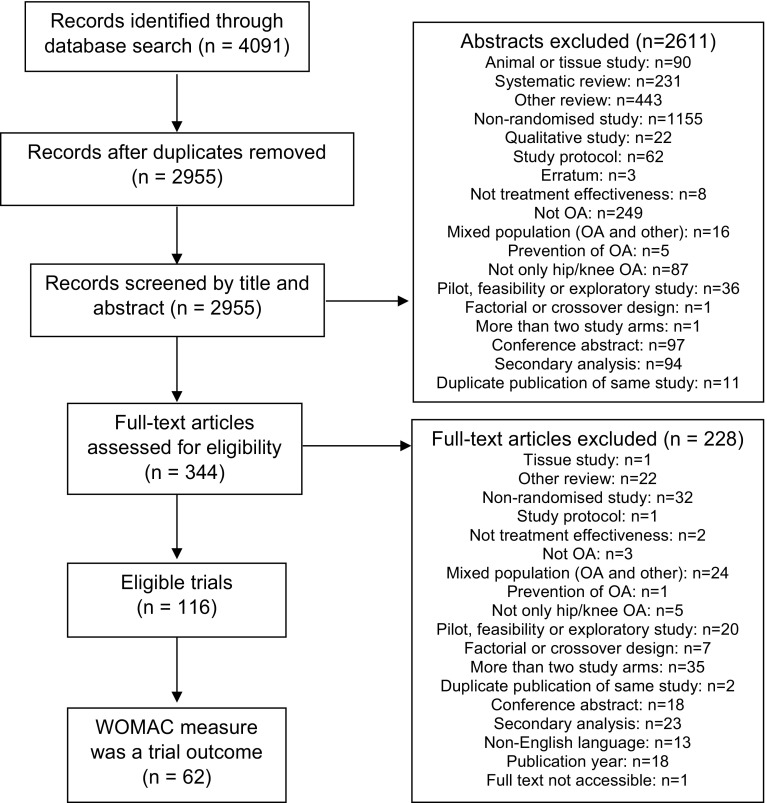
Flow of studies

The original cohort of trials included 116 studies, which were two-arm randomised trials of hip and/or knee osteoarthritis (Fig. 1). Of these 116 trials, 62 reported using the WOMAC as an outcome measure and were included in this review. The majority of the included trials were single-centre, superiority trials of knee osteoarthritis (Table 2). The median sample size was 75 randomised participants (IQR 50–148). The smallest trial randomised 20 participants and the largest randomised 606 participants. The median follow-up period was 4.5 months (IQR 1.5–6). Some trials assessed participants only immediately after a single session of an intervention. The maximum length of the follow-up period was 3 years.

**Table 2:** Characteristics of included studies (*n* = 62)

	*n*	%
Study design		
Individually randomised	61	98
Cluster-randomised	1	2
Study hypothesis		
Superiority	40	65
Non-inferiority	3	5
Multiple	1	2
Unclear	18	29
Allocation ratio		
1:1	61	98
Unclear	1	2
Study centres		
Single centre	46	74
Multi-centre	9	15
Unclear	7	11
Funding source		
Industry	8	13
Non-industry	25	40
Combination	3	5
No funding	4	6
Not reported	22	35
Population		
Knee OA	57	92
Hip OA	4	6
Hip or knee OA	1	2
Intervention		
Drug	19	31
Surgery	4	6
Exercise	14	23
Other	25	40
Comparator		
Active treatment	41	66
Usual care	8	13
Placebo or sham	13	21

**Fig. 2:**
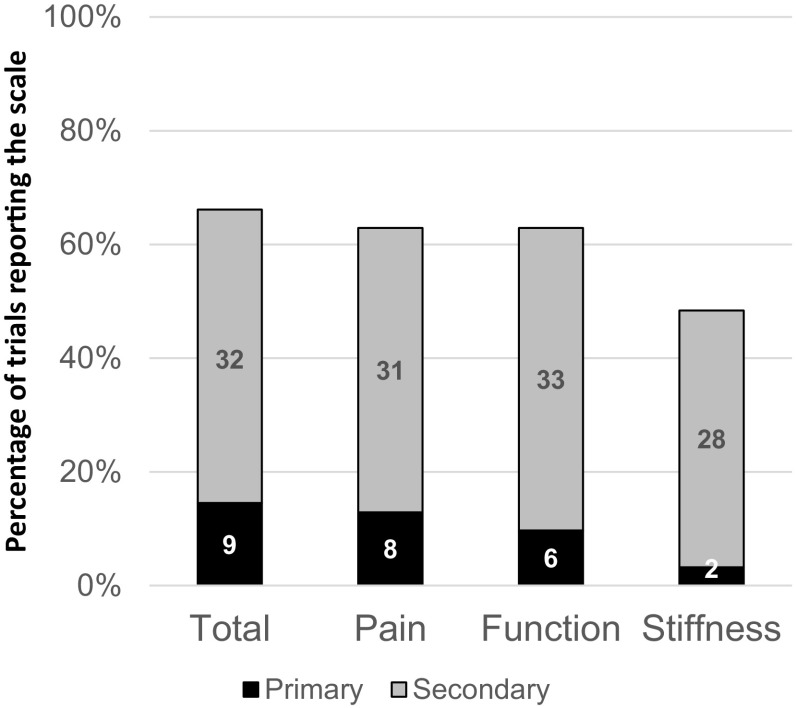
Use of WOMAC total and subscales as a trial outcome measure (*n* = 62) (Number of trials shown in graph bars, percentage of trials shown on y-axis)

The WOMAC total score was reported in 41 trials (66%, *n* = 41/62) and it was used as the primary outcome in 22% of these trials (*n* = 9/41) (Fig. 2). The pain and function subscales were reported in 39 trials (63%, *n* = 39/62). However, only 30 trials reported the stiffness subscale (48%, *n* = 30/62). Of the 41 trials reporting the WOMAC total score, less than half reported the results for all three individual subscales (pain, stiffness and function) (37%, *n* = 15/41) (Table 3).

**Table 3:** Reporting of WOMAC total and individual subscales (*n* = 62)

	*n* (%)
Reported total score	41 (66)
Total and all three subscales	15 (24)
Total and two subscales (pain and function)	4 (6)^a^
Total only	22 (35)
Did not report total score	21 (34)
Three individual subscales	15 (24)
Two subscales (pain and function)	4 (6)
One subscale	2 (3)^b^

^a^For one study, the total score used was the sum of the pain and function subscales only. For three studies, the stiffness subscale was used to calculate the total score but results on the stiffness subscale were not reported separately

^b^One study reported pain subscale only and one study reported the function subscale only

Few trials restricted eligibility of the trial participants using the WOMAC measure (6%, *n* = 4/62). For example, one trial only included participants > 300 on a 0–500 scale for the WOMAC pain subscale.

An alternative version of the WOMAC was used in 12 trials, most commonly using a translated version (*n* = 8), a shortened version with reduced items or combining only two subscales (*n* = 3) or alternative item weightings (*n* = 1). None of the trials made the WOMAC questionnaire available or provided details of the wording of individual items as part of the results publication. Of the 10 trials that referred to a published protocol, one trial provided the WOMAC questionnaire used as an appendix to the study protocol [31].

### Scoring of the WOMAC

The version of the WOMAC most commonly used was 5-point Likert scale with a range of 0–96 (41%, *n* = 17/41) (Table 4). The smallest range used was 0–10 and the largest range used was 0–2400.

**Table 4:** Version used and scoring of the WOMAC total (*n* = 41)

	*n*	%
Scoring version		
Likert scale	17	41
NRS	3	7
VAS	3	7
Unclear	18	44
Item range		
0–4	21	51
0–10	7	17
0–100	2	5
1–4	1	2
Unclear	10	24
Method to combine subscales		
Sum	31	76
Average	3	7
Sum and convert to percentage	1	2
Sum with equal weighting for subscales	1	2
Unclear	5	12

In most trials, the range for the total score was not reported and it was unclear how the individual items were combined. The range of the total score was often assumed from the mean score or range of a single item; however, it was still unclear for 20% of trials (*n* = 8/41).

For the individual WOMAC subscales, the scoring version and item range were reported more clearly than for the WOMAC total score. For example, the scoring version was unclear for 44% of trials reporting the WOMAC total score (*n* = 18/41), compared to 21% reporting the pain subscale (*n* = 8/39). Around ¾ of trials reported the version used and item range for each subscale (Online Resource 2). The range for the subscale was apparent for all trials; half of trials reported the range and for the other half, the range could be assumed based on the mean score or the item range.

In the trial results, four trials dichotomised participants based on the WOMAC total score (*n* = 2) or WOMAC pain score (*n* = 2) and none used the same cut-off points. Two additional studies categorised participants as ‘treatment responders’ using a combination of the WOMAC post-treatment score, WOMAC change score and the patient global assessment score according to the OARSI criteria [32]. No studies reported the WOMAC as a subset of the items from the KOOS score.

Trials reported that the WOMAC was completed by the participant (34%, *n* = 21/62) or by the participant with a clinician assessor (34%, *n* = 21/62). The data collection for the WOMAC was unclear for the remaining 20 studies (32%, *n* = 20/62). No trial reported that the WOMAC was collected by proxy for the participant. When an outcome assessor was involved, it was often unclear whether the participant completed a written version of the WOMAC questionnaire that was checked by the assessor or whether the assessor delivered the questionnaire verbally. One trial noted that ‘the questionnaire was read by the investigator for illiterate patients’ [33].

### Analysing the WOMAC

To analyse the WOMAC measure, the most commonly used methods were a *t* test, repeated-measures ANOVA or a mixed effects model (Table 5). The majority of trials used complete case analysis (24%, *n* = 10/41) and few trials used multiple imputation to assess the impact of missing data (7%, *n* = 3/41). Statistical analysis methods and handling of missing data was similar for the WOMAC total score and the individual subscales.

**Table 5:** Analysis of the WOMAC total score (*n* = 41)

	*n*	%
Statistical analysis method		
*t* test	13	33
Repeated-measures ANOVA	9	23
Mixed model	8	20
ANCOVA	3	8
Mann–Whitney *U* test	6	15
Other: Kruskal–Wallis *H* test	1	3
Adjusted for covariates		
Yes	11	27
Unclear	4	10
No	26	63
Method to handle missing data		
Complete case	10	24
Multiple imputation	3	7
Single imputation (e.g. LVCF)	3	7
Mixed model without imputation	5	12
No missing data	4	10
Unclear	16	39

For the WOMAC total score, around ¼ of trials reported using covariate adjustment in the analysis (27%, *n* = 11/41). Covariate adjustment was more common for analysis on the individual WOMAC subscales (46% for the pain subscale, *n* = 18/39). For adjusted analyses, trials most commonly adjusted for baseline score (13 trials), BMI (7 trials), sex (6 trials) and age (5 trials).

### Interpreting results from the WOMAC

The majority of studies reported the within-group mean post-treatment score (88%, *n* = 36/41 for WOMAC total) and the corresponding standard deviation (68%, *n* = 28/41 for WOMAC total) (Table 6). For the WOMAC total, the between-group mean difference with the corresponding 95% confidence interval was reported in less than 1/3 of trials (29%, *n* = 12/41). The proportion of studies reporting the between-group difference was higher for the WOMAC subscales but still remained below half (46%, *n* = 18/39 for the pain and function subscales). For the majority of trials, the results of the between-group analyses were reported using only the *p* value (49%, *n* = 20/41).

**Table 6:** Reporting of results for the WOMAC total score (*n* = 41)

	*n*	%
Summary score		
Mean post-treatment score	28	68
Mean change score	2	5
Mean post-treatment and change scores	8	20
Median post-treatment score	1	2
Multiple reported	1	3
None reported	1	3
Within-group variation		
Standard deviation	25	61
95% confidence interval	5	12
Range	1	2
Standard deviation and 95% confidence interval	2	5
Multiple reported	1	2
None reported	7	17
Between-group score		
Mean difference	13	32
None reported	28	68
Between-group variation		
95% confidence interval and *p* value	10	24
95% confidence interval	3	7
*p* value	20	49
*p* value interval (e.g. *p* < 0.05)	6	15
None reported	2	5

Of the 4 trials where dichotomisation of the WOMAC score was used, all reported the proportion of participants achieving the cut-off score, one reported the odds ratio and two reported the corresponding *p* value.

## Discussion

### Summary of findings

The WOMAC is a commonly used outcome measure in trials of hip and knee osteoarthritis. It was used to assess self-reported patient outcomes in around half of hip and knee osteoarthritis trials, predominantly as a secondary outcome measure. However, there was substantial variation in the way the WOMAC measure was implemented and analysed. The scoring range for the WOMAC total score was 0–96 for most trials; however, it was as small as 0–10 for some trials and as large as 0–2400 for others. Randomised trials often did not adequately report the version of the WOMAC used, how individual item scores were combined and the range of the score.

Most studies used a *t* test, repeated-measures ANOVA or mixed effects model to analyse the WOMAC score. The majority of studies did not adjust for baseline covariates in their analysis. In the study results, the mean score and variation within the treatment groups was well reported. However, interpretation of the results of between-group analyses was hindered by poor reporting, with less than half of trials reporting the between-group mean difference for the WOMAC score.

### Comparison with existing literature

Only one previous review has looked at the use of the WOMAC [3]. The results of this review align with and build upon the findings of Woolacott et al. Both reviews found that the type of scale and the score range were unclear for 20% and 10% of trials, respectively, with these values being explicitly reported in only around half of trials. Despite the passage of time and increasing awareness of, and initiatives to address, the need for improvement in the reporting of clinical trials, we found little evidence of improvement in reporting on essential characteristics of the WOMAC measure. There was also no indication of improvement in the consistency across trials on how the WOMAC is measured. Additionally, restricting focus to a particular clinical condition did not lead to greater consistency being observed. This suggests that the previously observed variation in implementation and poor reporting is a generic issue, irrespective of the clinical condition.

There were some differences between this review’s findings and that of Woolacott and colleagues. In this review, most trials reporting the WOMAC pain subscale also reported the function and stiffness subscales, whereas the previous review found that the function subscale was rarely reported. It is unclear whether this is due to changes over time or the differences of the interventions and clinical area being considered. However, both reviews found that many trials report the results using the WOMAC total score without reporting the results of the component subscales.

### Strengths and limitations

This review examines the reporting of the WOMAC in an up-to-date sample of trials identified using a systematic search strategy. This is the first review to examine the reporting of the function and stiffness subscales of the WOMAC, as well as the pain subscale and total score. This review did not restrict eligibility based on the intervention, including trials of physical therapies, surgery and pharmacological treatments.

This review is limited in that it only includes trials published during a 1-year period. Therefore, the results do not provide information on the trends in the use and reporting of the WOMAC over time. It also does not consider the use of the WOMAC in trials of conditions other than knee or hip osteoarthritis, such as ankle osteoarthritis [34, 35] or rheumatoid arthritis [7]. An additional limitation is that the use of the WOMAC may be different in observational or multi-arm studies, compared to two-arm randomised trials.

### Implications

This review demonstrates that problems persist in the poor reporting and inconsistency in the measurement of the WOMAC total score and the WOMAC subscale scores. This makes it difficult to interpret trial results and hinders comparisons between trials, for instance, where the range of the scale is unknown. The clinical importance of a 20-point difference between treatments varies greatly depending on whether a 0–96 scale or 0–2400 scale was used. Poor reporting of the effect estimates from between-group analyses could also hinder the interpretation of study results.

Analysing only the WOMAC total score without examining the individual subscales may hide intricacies in the study results. For example, there may be an improvement in pain but worsening function, which would not be evident since the total score remains unchanged. The presentation of the individual subscale results would be important if the changes in specific domains are seen as more desirable than others due to patient preference or the hypothesised treatment mechanisms [36]. Reporting the results of individual subscales also makes it easier to compare the trial results with other trials where only individual subscales are reported without the total score.

### Future research

Because there are multiple versions of the WOMAC measure in use, this means that research into the measurement properties of the WOMAC can be of uncertain applicability. Although the different versions have been shown to be correlated, it is unclear whether the psychometric properties are similar [37]. When assessing validity and responsiveness, the sensitivity of a 0–4 Likert scale, 0–10 NRS and 0–100 VAS are likely to be different. Future research should compare the different versions of the WOMAC measure, in terms of the validity, responsiveness and usability. The findings of this research could be used to make recommendations on which version of the WOMAC measure is most appropriate for particular settings and how it should be measured and analysed.

Further studies could also examine whether, or when, it is appropriate to ‘translate’ the results from one version of the WOMAC into another version. For instance, it is unclear whether results for the WOMAC Likert pain subscale (range 0–20) can be multiplied to translate the results onto the WOMAC VAS pain subscale (range 0–500). If it is appropriate to standardise different versions of the WOMAC to the same scale (e.g. converting all to a 0–100 scale), this would make it easier to compare the results of different trials and combine results in meta-analyses.

### Recommendations for using the WOMAC

When publishing the results of osteoarthritis trials, trialists should clearly report the version of the WOMAC measure that was used and the associated score range. Unless there is sufficient justification to do otherwise, trialists should favour the 5-point Likert scale version and combine scores by summation of the individual items. Since the Likert scale version is the most commonly used, this would make it easier to compare the results with the findings of previous trials.

Trialists using the WOMAC total score should also report results for the individual subscales for pain, function and stiffness. This would allow readers to see whether the treatment effect (or lack of it) is consistent across the three domains.

Trialists should also assess the robustness of the results based on adjustment for baseline covariates and the handling of missing data.

## Conclusion

While the WOMAC measure is commonly used in trials of hip and knee osteoarthritis, there is wide variation on how the WOMAC is implemented and analysed. Relevant details are often poorly reported and, as such, the ranges of the outcome scales are often unclear. This inhibits the interpretation of findings and comparisons with other studies. The interpretation of results on the WOMAC measure was also hindered by other limitations, such as not using an analysis method that estimates an effect size, not reporting an effect size estimate or not exploring the effects of assumptions on missing data mechanisms.

Trials should report the version of the WOMAC used, the score range and how item scores were combined. Future research should examine which version of the WOMAC measure has optimal properties. Improved consistency and transparency in how the WOMAC is measured would make it easier to interpret trial results and facilitate the comparison of results across trials.

## Electronic supplementary material

Below is the link to the electronic supplementary material.

Supplementary material 1 (DOCX 23 KB)

Supplementary material 2 (XLSX 33 KB)
