# Supplementary material for: Problems persist in reporting of methods and results for the WOMAC measure in hip and knee osteoarthritis trials
Source: Qual Life Res. 2018 Sep 18;28(2):335–43. doi: 10.1007/s11136-018-1978-1 (PMC6373321; doi:10.1007/s11136-018-1978-1)
Supplement: Supplementary file 1 — Supplementary material 1 (DOCX 23 KB) [file 11136_2018_1978_MOESM1_ESM.docx]

**Supplementary material contents:**

**Online resource 1. Search strategy example (MEDLINE Ovid)**

**Online resource 2. Tables for measurement of WOMAC subscales**

Table A1. Measurement of the WOMAC subscales

Table A2. Analysis of the WOMAC subscales

Table A3. Reporting of the WOMAC subscale results

**Article information:**

Article title: Problems persist in reporting of methods and results for the WOMAC measure in hip and knee osteoarthritis trials

Journal: Quality of Life Research

**Authors:** Copsey B ^1^, Thompson J ^1^, Vadher K ^1^, Ali U ^1^, Dutton SJ ^1^, Fitzpatrick R ^2^, Lamb SE ^1^, Cook JA ^1^.

^1^ Nuffield Department of Orthopaedics, Rheumatology and Musculoskeletal Sciences (NDORMS), University of Oxford, Botnar Research Centre, Windmill Road, Headington, Oxford, OX3 7LD.

^2^ Nuffield Department of Population Health, University of Oxford, Richard Doll Building, Old Road Campus, Oxford, OX3 7LF.

Corresponding author (BC): Email: [bethan.copsey@csm.ox.ac.uk](mailto:bethan.copsey@csm.ox.ac.uk), tel: +44 (0)1865 737923.

**Online resource 1. Search strategy example (MEDLINE Ovid)**

1 randomized controlled trial.pt.

2 controlled clinical trial.pt.

3 randomized.ab.

4 placebo.ab.

5 clinical trial/

6 randomly.ab.

7 trial.ti.

8 1 or 2 or 3 or 4 or 5 or 6 or 7

9 humans/

10 8 and 9

11 exp Osteoarthritis/

12 osteoarthr\$.tw.

13 (degenerative adj3 (arthr\$ or joint\$ or disease\$)).tw.

14 arthros?s.tw.

15 11 or 12 or 13 or 14

16 10 and 15

17 limit 16 to yr="2016"

**Online resource 2. Tables for measurement of WOMAC subscales**

Table A1. Measurement of the WOMAC subscales

|  | **Pain** | | **Function** | | **Stiffness** | |
| --- | --- | --- | --- | --- | --- | --- |
|  | n | % | n | % | n | % |
| **Scoring version** |  |  |  |  |  |  |
| Likert scale | 24 | 62% | 24 | 62% | 18 | 60% |
| NRS | 3 | 8% | 3 | 8% | 1 | 3% |
| VAS | 4 | 10% | 4 | 10% | 4 | 13% |
| Unclear | 8 | 21% | 8 | 21% | 7 | 23% |
| **Item range** |  |  |  |  |  |  |
| 0-4 | 25 | 64% | 25 | 64% | 19 | 63% |
| 0-10 | 7 | 18% | 7 | 18% | 5 | 17% |
| 0-100 | 5 | 13% | 5 | 13% | 5 | 17% |
| Unclear | 2 | 5% | 2 | 5% | 1 | 3% |
| **Maximum of scale** |  |  |  |  |  |  |
| Median (IQR) | 20 (20-50) | | 68 (68-150) | | 8 (8-20) | |
| Range | 10-500 | | 10-1700 | | 8-200 | |

Table A2. Analysis of the WOMAC subscales

|  | **Pain** | | **Function** | | **Stiffness** | |
| --- | --- | --- | --- | --- | --- | --- |
|  | n | % | n | % | n | % |
| **Statistical analysis method** |  | | | | | |
| t-test | 11 | 28% | 11 | 28% | 10 | 33% |
| Repeated measures ANOVA | 7 | 18% | 8 | 21% | 6 | 20% |
| Mixed model | 10 | 26% | 10 | 26% | 7 | 23% |
| Linear regression adjusted for baseline WOMAC score (ANCOVA) | 5 | 13% | 4 | 10% | 4 | 13% |
| Mann Whitney U-test | 4 | 10% | 4 | 10% | 3 | 10% |
| Other^a^ | 2 | 5% | 2 | 5% | 0 | 0% |
| **Adjusted for covariates** |  | | | | | |
| Yes | 18 | 46% | 17 | 44% | 12 | 40% |
| Unclear | 0 | 0% | 0 | 0% | 0 | 0% |
| No | 21 | 54% | 22 | 56% | 18 | 60% |
| **Method to handle missing data** |  | | | | | |
| Complete case | 10 | 26% | 10 | 26% | 7 | 23% |
| Multiple imputation | 5 | 13% | 5 | 13% | 4 | 13% |
| Single imputation (e.g. LVCF) | 5 | 13% | 5 | 13% | 3 | 10% |
| Mixed model without imputation | 5 | 13% | 5 | 13% | 3 | 10% |
| No missing data | 2 | 5% | 2 | 5% | 2 | 7% |
| Unclear | 12 | 31% | 12 | 31% | 11 | 37% |

^a^ Other analysis techniques were described as: Generalised estimating equation (GEE) model (n=1) and general linear model (n=1) for the pain and function subscales

Table A3. Reporting of the WOMAC subscale results

|  | **Pain** | | **Function** | | **Stiffness** | |
| --- | --- | --- | --- | --- | --- | --- |
|  | **n** | **%** | **n** | **%** | **n** | **%** |
| **Summary score** |  | | | | | |
| Mean post-treatment score | 18 | 46% | 20 | 51% | 15 | 50% |
| Mean change score | 3 | 8% | 3 | 8% | 3 | 10% |
| Mean post-treatment and change scores | 13 | 33% | 12 | 31% | 7 | 23% |
| Median post-treatment score | 2 | 5% | 2 | 5% | 2 | 5% |
| Median post-treatment and change scores | 0 | 0% | 0 | 0% | 2 | 5% |
| Multiple reported | 2 | 5% | 1 | 3% | 1 | 3% |
| None reported | 1 | 3% | 1 | 3% | 0 | 0% |
| **Within-group variation** |  | | | | | |
| Standard deviation | 22 | 56% | 22 | 56% | 17 | 57% |
| Standard error | 1 | 3% | 1 | 3% | 0 | 0% |
| 95% confidence interval | 6 | 15% | 6 | 15% | 4 | 13% |
| Range | 0 | 0% | 0 | 0% | 1 | 3% |
| Standard deviation and 95% confidence interval | 4 | 10% | 4 | 10% | 4 | 13% |
| Interquartile range | 1 | 3% | 1 | 3% | 1 | 3% |
| Multiple reported | 1 | 3% | 1 | 3% | 1 | 3% |
| None reported | 4 | 10% | 4 | 10% | 2 | 7% |
| **Between-group effect size** |  | | | | | |
| Mean difference | 18 | 46% | 18 | 46% | 12 | 40% |
| None reported | 21 | 54% | 21 | 54% | 18 | 60% |
| **Between-group comparison** |  | | | | | |
| 95% confidence interval and p-value | 12 | 31% | 12 | 31% | 10 | 33% |
| 95% confidence interval | 5 | 13% | 5 | 13% | 2 | 7% |
| Exact p-value | 19 | 49% | 18 | 46% | 16 | 53% |
| p-value category (e.g p<0.05) | 3 | 8% | 3 | 8% | 2 | 7% |
| None reported | 0 | 0% | 1 | 3% | 0 | 0% |
